# Assessing the progression of chronic periodontitis using subgingival pathogen levels: a 24-month prospective multicenter cohort study

**DOI:** 10.1186/s12903-017-0337-x

**Published:** 2017-01-16

**Authors:** E. Kakuta, Y. Nomura, T. Morozumi, T. Nakagawa, T. Nakamura, K. Noguchi, A. Yoshimura, Y. Hara, O. Fujise, F. Nishimura, T. Kono, M. Umeda, M. Fukuda, T. Noguchi, N. Yoshinari, C. Fukaya, S. Sekino, Y. Numabe, N. Sugano, K. Ito, H. Kobayashi, Y. Izumi, H. Takai, Y. Ogata, S. Takano, M. Minabe, A. Makino-Oi, A. Saito, Y. Abe, S. Sato, F. Suzuki, K. Takahashi, T. Sugaya, M. Kawanami, N. Hanada, S. Takashiba, H. Yoshie

**Affiliations:** 1 Department of Oral Microbiology, Tsurumi University School of Dental Medicine, 2-1-3 Tsurumi, Tsurumi-ku, Yokohama, Japan; 2 Department of Translational Research, Tsurumi University School of Dental Medicine, 2-1-3 Tsurumi, Tsurumi-ku, Yokohama, 230-8501 Japan; 3 Division of Periodontology, Department of Oral Biological Science, Niigata University Graduate School of Medical and Dental Sciences, 2-5274 Gakkocho-dori, Chuo-ku, Niigata Japan; 4 Department of Dentistry and Oral Surgery, School of Medicine, Keio University, 35 Shinano-machi, Shinjuku-ku, Tokyo, Japan; 5 Department of Periodontology, Kagoshima University Graduate School of Medical and Dental Sciences, 8-35-1 Sakuragaoka, Kagoshima, Japan; 6 Department of Periodontology, Unit of Translational Medicine, Nagasaki University Graduate School of Biomedical Sciences, 1-12-4 Sakamoto, Nagasaki, Japan; 7 Section of Periodontology, Division of Oral Rehabilitation, Faculty of Dental Science, Kyushu University, 3-1-1 Maidashi, Higashi-ku, Fukuoka, Japan; 8 Department of Periodontology, Osaka Dental University, 8-1 Kuzuhahanazonocho, Hirakata, Japan; 9 Department of Periodontology, School of Dentistry, Aichi Gakuin University, 2-11 Suemori-doori,Chikusa-ku, Nagoya, Japan; 10 Department of Periodontology, School of Dentistry, Matsumoto Dental University, 1780 Hirokagobara, Shiojiri, Nagano Japan; 11 Department of Periodontology, School of Life Dentistry at Tokyo, The Nippon Dental University, 1-9-20 Fujimi, Chiyoda-ku, Tokyo, Japan; 12 Department of Periodontology, Nihon University School of Dentistry, 1-8-13 Kanda-Surugadai, Chiyoda-ku, Tokyo, Japan; 13 Department of Periodontology, Graduate School of Medical and Dental Sciences, Tokyo Medical and Dental University, 1-5-45 Yushima, Bunkyo-ku, Tokyo, Japan; 14 Department of Periodontology, Nihon University School of Dentistry at Matsudo, 2-870-1 Sakae-cho-nishi, Matsudo-shi, Chiba Japan; 15 Bunkyo-Dori Dental Clinic, 2-4-1 Anagawa, Inage-ku, Chiba, Japan; 16 Division of Periodontology, Department of Oral function and Restoration, School of Dentistry, Kanagawa Dental University, 82 Inaokacho, Yokosuka, Kanagawa Japan; 17 Department of Periodontology, Tokyo Dental College, 2-9-18 Misakicho, Chiyoda-ku, Tokyo, Japan; 18 Comprehensive Dental Care, The Nippon Dental University Niigata Hospital, 1-8 Hamaura-cho, Chuo-ku, Niigata, Japan; 19 Department of Periodontology, School of life Dentistry at Niigata, The Nippon Dental University, 1-8 Hamaura-cho, Chuo-ku, Niigata, Japan; 20 Division of Dental Anesthesiology, Department of Oral Surgery, School of Dentistry, Ohu University, 31-1 Misumido, Tomita, Koriyama, Fukushima Japan; 21 Division of Periodontics, Department of Conservative Dentistry, School of Dentistry, Ohu University, 31-1 Misumido, Tomita, Koriyama, Fukushima Japan; 22 Division of Periodontology and Endodontology, Department of Oral Health Science, Hokkaido University Graduate School of Dental Medicine, Kita 13, Nishi 7, Kita-ku, Sapporo, Japan; 23 Department of Pathophysiology-Periodontal Science, Okayama University Graduate School of Medicine, Dentistry and Pharmaceutical Sciences, 2-5-1 Shikata-cho, Okayama, Japan

**Keywords:** Progression of periodontitis, Periodontal stability, *Porphyromonas gingivalis*, Subgingival plaque

## Abstract

**Background:**

The diagnosis of the progression of periodontitis presently depends on the use of clinical symptoms (such as attachment loss) and radiographic imaging. The aim of the multicenter study described here was to evaluate the diagnostic use of the bacterial content of subgingival plaque recovered from the deepest pockets in assessing disease progression in chronic periodontitis patients.

**Methods:**

This study consisted of a 24-month investigation of a total of 163 patients with chronic periodontitis who received trimonthly follow-up care. Subgingival plaque from the deepest pockets was recovered and assessed for bacterial content of *Porphyromonas gingivalis*, *Prevotella intermedia*, and *Aggregatibacter actinomycetemcomitans* using the modified Invader PLUS assay. The corresponding serum IgG titers were measured using ELISA. Changes in clinical parameters were evaluated over the course of 24 months. The sensitivity, specificity, and prediction values were calculated and used to determine cutoff points for prediction of the progression of chronic periodontitis.

**Results:**

Of the 124 individuals who completed the 24-month monitoring phase, 62 exhibited progression of periodontitis, whereas 62 demonstrated stable disease. The *P. gingivalis* counts of subgingival plaque from the deepest pockets was significantly associated with the progression of periodontitis (*p* < 0.001, positive predictive value = 0.708).

**Conclusions:**

The *P. gingivalis* counts of subgingival plaque from the deepest pockets may be associated with the progression of periodontitis.

**Electronic supplementary material:**

The online version of this article (doi:10.1186/s12903-017-0337-x) contains supplementary material, which is available to authorized users.

## Background

Periodontitis is a chronic inflammation, which is leaded by periodontal bacteria and host immune response [1]. Periodontitis results in loss of connective tissue and bone support and is a major cause of tooth loss in adults [2]. The maintenance care following the initial active therapy phase plays an essential part in periodontal treatment to prevent disease progression based on the patient’s individual needs [3].

The clinical diagnosis of chronic periodontal disease is based on visual and radiographic assessment of the periodontal tissues and on measurements of the space between the tooth and gum [2]. However, traditional clinical criteria are often insufficient for determining sites of active disease, for monitoring quantitatively the response to therapy or for measuring the degree of susceptibility to future disease progression [4]. Therefore, it is necessary to establish effective markers indicating periodontitis progression.

The role of salivary biomarkers and periodontal pathogens were determined during periodontal disease progression [5]. Salivary *Porphyromonas gingivalis* ratio (ratio: *P. gingivalis* counts/Total bacteria counts) may be potential indicators for the progression of periodontitis. Such a salivary test could be a useful diagnostic tool for predicting periodontal disease progression [6]. And also, our previous report suggested the *P. gingivalis* ratio would be an indicator for the progression of periodontitis [7]. Therefore, salivary periodontal pathogens were suggested as useful marker of periodontitis progression.

While, several previous studies determined the subgingival plaque pathogens were useful indicators of periodontal progression. Monitoring the proportions of *P. gingivalis* and *Treponema denticola* in subgingival plaque had the potential to help identify sites at significant risk for progression of periodontitis [8]. The levels of red complex bacteria (*P. gingivalis*, *Tannerella forsythia*, and *T. denticola*) in subgingival plaque had a strong relationship with the clinical parameters [9]. Higher levels of *P. gingivalis*, *Prevotella intermedia* and *Aggregatibacter actinomycetemcomitans* in subgingival plaque appear to be related to an increased risk of periodontitis progression [10–12]. Similarly, *A. actinomycetemcomitans*, *P. gingivalis*, and *P. intermedia* may be useful as indicators of periodontitis activity [13]. In addition, presence of *A. actinomycetemcomitans* in subgingival plaque was a risk marker for periodontitis progression at the full mouth level [14, 15]. The increase in the number of *P. gingivalis*, *P. intermedia* in subgingival plaque of adolescents depends on the severity of gingivitis and appears to be a contributing factor for the shift from gingivitis to periodontitis [16, 17]. Thus, periodontal pathogens of subgingival plaque were valuable risk markers for periodontitis progression.

The bacterial tests or serum IgG tests were used to monitor periodontitis progress [5, 6, 18]. However, it remains unclear what periodontal bacteria from subgingival plaque are the most useful for estimation of periodontitis progression during regular follow-ups after initial therapy. In addition, previous reports did not test for possible correlations between the levels of periodontal bacteria in saliva and those in subgingival plaque from the deepest pockets.

The aims of this multicenter study were two-fold. The first goal was to evaluate subgingival bacteriological markers among patients treated for chronic periodontitis who exhibited or lacked subsequent disease progression during 24 months of regular follow-ups. The second goal was to define diagnostic values for indicating periodontitis progression and stability, including comparison between the levels of periodontal pathogens in subgingival plaque from the deepest pockets compared to those in saliva.

## Methods

### Study design

This study was performed as a clinical research project for diagnosis of periodontitis, as sponsored by the Japanese Society of Periodontology. Subjects were recruited in cooperation with 17 facilities (16 university hospitals and one clinic) in Japan, and were registered between February 2009 and February 2012. An additional file shows this in more detail [see Additional file 1]. The study enrolled a total of 163 follow-up patients, that is, individuals with chronic periodontitis who had completed active treatments such as initial therapy or periodontal surgery. Each diagnosis was based on the Guidelines of the American Academy of Periodontology [19]. All individuals were of age ≥ 30 years, systemically healthy, possessed at least 20 teeth, and had not taken systemic antibiotics, anti-inflammatory drugs, or immunosuppressive drugs within 3 months prior to enrollment.

### Clinical assessment

The follow-up patients were seen trimonthly over a 24-month period. At each visit, the patients received treatment only to remove supragingival plaque and calculus, if detected. To identify disease progression, a full-mouth periodontal examination, except for the third molars, was performed every 6 months. The following clinical parameters were recorded: plaque index (PlI), bleeding on probing (BOP), probing pocket depth (PPD), and clinical attachment level (CAL). PlI was recorded at 4 sites per tooth (mesial, buccal, distal, and lingual). PPD, CAL, and BOP were recorded at 6 sites per tooth (mesiobuccal, buccal, distobuccal, mesiolingual, lingual, and distolingual). Patients with at least one site of CAL loss of ≥3 mm at a given site over the 24-month study period were considered to be exhibiting periodontitis progression [6, 7, 20, 21]. Intra- and inter-examiner calibration sessions were conducted at the beginning and middle of the study period.

### Sample collection

At baseline, samples of subgingival plaque from the deepest pockets, saliva, and blood were collected from each patient. Samplings were carried out according to the following order; blood samples, saliva samples, and subgingival plaque samples. Whole saliva was collected from each subject by having the individual chew on a gum base, containing neither fragrance nor flavored ingredients, for 5 min. The subgingival plaque was collected as follows: after removal of the supragingival plaque, samples of subgingival plaque from the deepest pockets (except for those at the third molars) were obtained by consecutive insertion (at a given site) of two sterile number-40 paper points (Zipperer Absorbent Paper Points, VDW GmbH, Munich, Germany) into the periodontal pocket for 10 s per point [22]. The plaque and saliva samples were sent immediately to a medical laboratory (BML Corporation, Tokyo, Japan) for bacterial analysis [6, 17, 23]. Blood sampling was performed using a commercial kit (DEMECAL Blood Test Set; Sunstar Inc, Osaka, Japan). In brief, device-treated serum was obtained from a 50-*μ*L sample of whole capillary blood collected from the middle fingertip. The samples of sera were sent immediately to a commercial laboratory (Leisure Inc., Tokyo, Japan) for immune analysis [7, 24–27].

### Quantification of periodontal bacteria in subgingival plaque from the deepest pockets and in saliva

Quantitative analysis of the total bacterial count and periodontopathic bacterial counts, including *P. gingivalis*, *P. intermedia*, and *A. actinomycetemcomitans*, was performed using a modification of the Invader PLUS assay [7, 22, 28, 29]. Briefly, bacterial DNA was extracted from samples of the subgingival plaque from the deepest pockets by suspending each plaque sample in 1 mL of phosphate-buffered saline, pH 7.4 and processing using the MagNA Pure LC Total Nucleic Acid Isolation Kit (Roche, Basel, Switzerland) according to the manufacturer’s instructions. Similarly, bacterial DNA was extracted from the 100-*μ*l whole saliva samples using the MagNA Pure LC Total Nucleic Acid Isolation Kit. The individual sequences of each bacterial species were obtained from a public database (National Center for Biotechnology Information, Bethesda, MD). Primers for each species were designed based on a region of the 16S rRNA gene. A pair of universal primers and a universal probe were used to determine the total number of bacteria. Primary probes and Invader oligos were designed using the Invader technology creator (HOLOGIC, Madison, WI) and were based on sequences in the amplified regions [7, 28–30].

Template DNA was added to a 15-*μ*L reaction mixture containing primers for each species, 50 *μ*M dNTPs, 700 nM primary probe, 70 nM Invader oligo, 2.5 U polymerase chain reaction (PCR) enzyme (EagleTaq DNA polymerase, Roche, Basel, Switzerland), and the Invader core reagent kit (Cleavase XI Invader core reagent kit, HOLOGIC, Madison, WI) containing a fluorescence resonance energy transfer (FRET) mix and an enzyme/MgCl_2_ solution. The reaction mixture was preheated at 95 °C for 20 min, and a 2-step PCR reaction (95 °C for 1 s and 63 °C for 1 min) was performed for 35 cycles using an ABI PRISM 7900 thermocycler (Applied Biosystems, Foster City, CA). Fluorescence values of carboxyfluorescein (FAM; wavelength/bandwidth: excitation, 485/20 nm; emission, 530/25 nm) were measured at the end of the incubation/extension step at 63 °C for each cycle.

The limit of detection for this method was determined for each species with dilutions of bacterial DNA. Standard curves were constructed based on a crossing point determined by the fit point method.

Next, the proportions of the 3 pathogens within the total bacterial counts were determined [10, 31]. Bacterial ratios (%) and bacterial counts (log_10_) for each species also were used in various comparison and diagnostic analyses.

### Measurement of IgG titers against periodontal bacteria

Serum IgG antibody titers against periodontal pathogens were determined by Leisure Inc. using enzyme-linked immunosorbent assay (ELISA) [32]. The method and selection of bacterial antigens were based on previous reports [7, 24–27]. Briefly, sonicated preparations of *P. gingivalis* FDC381, *P. intermedia* ATCC25611, and *A. actinomycetemcomitans* ATCC29523 were used as representative bacterial antigens. Each antigen was diluted to 10 μg/mL with 0.1 M carbonate buffer (pH 9.6). An aliquot of this diluted solution (100 μL) then was added to each well in a flat-bottomed microtiter plate (Greiner Co. Ltd., Frickenhausen, Germany) and the plate was stored overnight at 4 °C. Each well with immobilized antigen was washed three times with PBS containing 0.05% Tween-20 (PBST). Subsequently, a diluted serum sample (3100-fold dilution with PBST) was added to each well. After incubation at 37 °C for 2 h, each well was washed three times with PBST and bound/free (B/F) separation was carried out. Next, a 100-μl aliquot of 1:5000 diluted alkaline phosphatase-conjugated goat antihuman IgG antibody (Jackson Immuno Research Laboratories, Inc., Baltimore, MD) was added to each well. After incubation at 37 °C for 2 h, each well was washed three times with PBST and B/F separation was carried out. Thereafter, 50 μl of *p*-nitrophenyl phosphate (Wako Pure Chemical Industries, Ltd., Osaka, Japan) adjusted to 1 mg/ml with 10% diethanolamine buffer (pH 9.8) was added to each well as substrate. The plate then was incubated at room temperature for 10–20 min. The enzymatic reaction was terminated by adding 50 μl of 3 M NaOH, and optical density (measurement at 405 nm; reference at 490 nm) was measured in a Micro ELISA Auto Reader (Bio-Rad Laboratories, Hercules, CA).

The standard titration curves were formulated using serial dilutions of pooled control serum collected from participants without periodontitis. The absorbance of each sample after reaction was defined as an ELISA unit (EU), such that 100 EU corresponded to 1:3200 dilution of the control sample. The antibody titers were expressed as standardized values calculated as follows: (EU for study serum samples—EU for control samples)/2 standard deviations (SD), using SDs as determined based on ELISA of the control samples [25].

### Statistical analysis

Descriptive analysis of the collected data was performed; the results are presented as medians. The Mann Whitney U test was used to compare clinical parameters, periodontal bacterial amounts, and IgG titers with or without progression of chronic periodontitis (CP). Only correlation with sex was performed using the Fisher’s exact test. To determine an optimal test for distinguishing follow-up patients with periodontal progression or stability, we constructed receiver operating characteristic (ROC) curves and set the cutoff point based on the minimum difference between sensitivity and specificity [6, 25]. On the basis of the calculated cutoff point, we then made appropriate cross-tabulations. Diagnostic values were calculated by the Fisher’s exact test to identify statistically significant differences. To check the correlation of periodontal bacteria with disease progression, logistic regression analysis was carried out to eliminate possible confounding variables. This study was originally designed to evaluate the diagnostic salivary levels of *P. gingivalis* in patients with or without the progression of CP. Calculation of the sample size was described in our previous report [7]. For the present study, post-hoc power analysis calculation using the cross table for patients with or without progression of CP against positive or negative for *P. gingivalis* suggested that groups would have to consist of at least 41 subjects each. A similar analysis revealed that groups of at least 26 subjects each would be required to detect differences in *P. gingivalis* counts (log_10_) in subgingival plaque from the deepest pockets. All analyses were performed by independent statisticians (Y.N., E.K., and N.H.) using IBM SPSS Statistics ver. 19.0 software (IBM Japan, Tokyo, Japan) and S-plus (ver. 6.0, NTT DATA, Tokyo, Japan).

## Results

Of the 163 follow-up patients enrolled in the study, 32 dropped out during the study period due to a variety of causes (including occurrence of the Great East Japan Earthquake), and 7 others withdrew because of the use during the study of antimicrobial agents for the treatment of acute periodontal abscesses. In the end, 124 subjects successfully completed the study protocol. Of the 124 patients who completed the study protocol, 62 follow-up patients (50%) presented with disease progression, whereas the same number of patients (50%) demonstrated stable disease.

Demographic characteristics and biological variables of the study participants at baseline are shown in Table 1. BOP, the deepest PPD, and the deepest CAL were significantly higher in subjects who exhibited progressive CP compared to patients with stable CP. For the pathogens, *P. gingivalis* counts in subgingival plaque from the deepest pockets, *P. intermedia* counts in subgingival plaque from the deepest pockets, *P. gingivalis* counts in saliva, and *P. intermedia* counts in saliva were significantly higher in subjects who exhibited progressive CP compared to patients with stable CP.

**Table 1:** Clinical parameters and bacterial levels at baseline in the patients with stable CP and progressive CP

	Stable CP patients (*n* = 62)	Progressive CP patients (*n* = 62)	*p*-value
Gender (male/female)	23/39	26/36	0.199
Age (year)	60.0 (56.0–66.0)	61 (52.5–68.0)	0.193
Smoking (non-smoker/smoker)	59/3	61/1	0.619
PlI	0.12 (0.06–0.30)	0.17 (0.07–0.45)	0.291
BOP (% positive)	3.62 (1.53–11.28)	6.54 (2.48–10.45)	0.022*
PPD (mm)	2.15 (1.80–2.53)	2.26 (1.76–2.57)	0.885
The deepest PPD (mm)	4.0 (4.00–6.00)	5.0 (4.25–7.75)	<0.001*
CAL (mm)	2.8 (2.34–3.33)	2.87 (2.78–3.62)	0.710
The deepest CAL (mm)	7.00 (5.25–8.00)	7.5 (6.25–9.00)	0.042*
*P. gingivalis* counts (log_10_) Subgingival plaque	1.00 (1.00–3.17)	1.64 (1.00–4.29)	<0.001*
*P. gingivalis* counts (log_10_) Saliva	2.59 (1.00–3.66)	3.34 (2.67–4.08)	0.002*
*P. gingivalis* IgG titers (EU)	1.28 (0.32–4.26)	1.63 (0.95–6.34)	0.503
*P. intermedia* counts (log_10_) Subgingival plaque	1.00 (1.00–1.63)	1.00 (1.00–2.72)	0.049*
*P. intermedia* counts (log_10_) Saliva	1.87 (1.00–3.65)	3.02 (1.41–4.05)	0.038*
*P. intermedia* IgG titers (EU)	−0.39 (0.47–0.13)	−0.27 (0.45–0.21)	0.173
*A. actinomycetemcomitans* counts (log_10_) Subgingival plaque	1.00 (1.00–1.00)	1.00 (1.00–1.00)	0.164
*A. actinomycetemcomitans* counts (log_10_) Saliva	1.00 (1.00–1.00)	1.00 (1.00–1.50)	0.065
*A. actinomycetemcomitans* IgG titers (EU)	−0.26 (0.41–0.03)	−0.30 (0.40–0.10)	0.278

Value represent median value (25–75 percentile). *CP* chronic periodontitis, *PlI* plaque index, *BOP* bleeding on probing, *PPD* probing pocket depth, *CAL* clinical attachment level, *EU* enzyme-linked immunosorbent assay unit

The deepest PPD and the deepest CAL indicate the value of the sites which is the deepest PPD and the deepest CAL in each subject, respectively. *p* values for gender were calculated using the Fisher’s exact test, and all others were Mann Whitneys’ U tests. The *mark represent statistical significance between stable CP patients and progressive CP patients (*p* < 0.05)

**Fig. 1:**
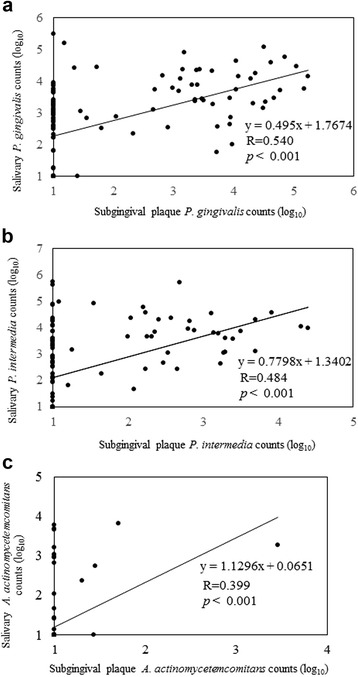
Scatter plots of periodontal pathogen counts from salivary and subgingival plaque from the deepest pockets for individual subjects. Correlations of periodontal pathogen counts in saliva and subgingival plaque from the deepest pockets were statistically significant. The correlation coefficient of bacterial counts in saliva and subgingival plaque from the deepest pockets was the highest for *P. gingivalis* (panel **a**) when compared with other pathogens (panel **b**, **c**). Note, for each of the three bacterial species, that bacterial counts were below the limit of detection in some subgingival plaque samples; this observation was most notable for *A. actinomycetemcomitans*, which could not be detected in >95% of samples (panel **c**).

Figure 1 shows the scatter plots of periodontal pathogen counts in saliva compared to those in subgingival plaque from the deepest pockets. Correlation coefficients of bacterial counts from saliva and subgingival plaque from the deepest pockets were 0.540 (*p* < 0.001) for *P. gingivalis*, 0.484 (*p* < 0.001) for *P. intermedia*, and 0.399 (*p* < 0.001) for *A. actinomycetemcomitans.* However, in some samples of subgingival plaque from the deepest pockets, bacterial counts were below the limit of detection. Notably, *A. actinomycetemcomitans* was not detected in 95.7% of samples of subgingival plaque from the deepest pockets.

To determine an optimal test for distinguishing follow-up patients with periodontal progression from those with stable CP, we constructed receiver operating characteristic curves and set the cutoff points on the basis of the minimum differences between sensitivity and specificity [6, 25]. Furthermore, on the basis of the cutoff point, we made appropriate cross-tabulations.

Table 2 shows the cutoff points, sensitivity, specificity, positive predictive value, negative predictive value, likelihood, and area under the curve for the periodontal bacterial counts and IgG titers. Among the 3 periodontal pathogens, only *P. gingivalis* yielded counts that demonstrated significant correlation with CP status (cutoff point = 1.370 (*p* < 0.001), sensitivity = 0.548, specificity = 0.774, positive predictive value = 0.708, negative predictive value = 0.632, likelihood ratio = 2.429, AUC = 0.666).

**Table 2:** Diagnostic values of subgingival plaque bacterial levels and serum IgG titers for periodontal bacteria

	Cutoff	*p*-value	Sensitivity	Specificity	Positive predictive value	Negative predictive value	Likelihood ratio	AUC
*P. gingivalis* counts (log_10_)	1.370	<0.001	0.548	0.774	0.708	0.632	2.429	0.666
*P. intermedia* counts (log_10_)	1.040	0.077	0.371	0.774	0.622	0.552	1.643	0.568
*A. actinomycetemcomitans* counts (log_10_)	1.151	0.171	0.065	0.984	0.800	0.513	4.000	0.478
*P. gingivalis* IgG titer^a^	1.355	>0.999	0.516	0.500	0.508	0.508	1.032	0.444
*P. intermedia* IgG titer^a^	−0.345	0.281	0.565	0.548	0.556	0.557	1.250	0.535
*A. actinomycetemcomitans* IgG titer^a^	−0.275	0.590	0.452	0.484	0.467	0.469	0.875	0.571

Fisher’s exact test was used for statistical analysis (*p* < 0.05). The *P. gingivalis* counts was statistically significant

The area under the curve of the *P. gingivalis* counts was the largest amongst all markers. Subgingival plaque *P. gingivalis* counts specificity was higher than salivary *P. gingivalis* counts (0.323) [7]

*EU* enzyme-linked immunosorbent assay unit, *AUC* area under the curve

^a^: J Periodontal Res. 2016 Dec;51(6):768–778

The area under the curve of the *P. gingivalis* counts was the largest amongst all markers evaluated. The specificity of *P. gingivalis* counts in subgingival plaque from the deepest pockets was higher than that for salivary *P. gingivalis* counts (0.323) [7].

To check the importance of periodontal bacteria in the progression of periodontal disease, logistic regression analysis was carried out to eliminate possible confounding variables. As shown in Table 3, the crude odds ratio of the deepest CAL in each subject, and *P. gingivalis* count were statistically significant. According to the multivariate adjusted odds ratio, only *P. gingivalis* counts exhibited a significant (*p* = 0.034) correlation with CP status.

**Table 3:** Multiple logistic regression analysis for progression of chronic periodontitis

	Crude OR (95% CI)	*p*-value	Multivariate adjusted OR (95% CI)	*p*-value
Gender (male/female)	0.62 (0.30–1.29)	0.200	1.06 (0.43–2.65)	0.893
Age (years)	1.02 (0.98–1.07)	0.271	1.03 (0.97–1.08)	0.324
PlI	2.15 (0.37–12.48)	0.393	0.88 (0.10–8.10)	0.909
BOP (% positive)	1.04 (1.00–1.08)	0.077	1.03 (0.98–1.08)	0.285
CAL (mm)	1.14 (0.69–1.87)	0.603	0.72 (0.33–1.58)	0.415
The deepest CAL (mm)	1.25 (1.01–1.54)	0.039*	1.35 (0.99–1.85)	0.059
*P. gingivalis* counts (log_10_)	4.163 (1.913–9.06)	<0.001*	1.56 (1.03–2.34)	0.034*
*P. gingivalis*IgG titers (EU)	1.01 (0.98–1.05)	0.422	1.01 (0.99–1.03)	0.276
*P. intermedia* counts (log_10_)	2.022 (0.92–4.443)	0.080	0.99 (0.52–1.76)	0.973
*P. intermedia* IgG titers (EU)	1.45 (0.46–4.60)	0.531	2.04 (0.50–8.22)	0.318
*A. actinomycetemcomitans* counts (log_10_)	4.207 (0.457–38.757)	0.205	44.70 (0.05–36,574)	0.267
*A. actinomycetemcomitans* IgG titers (EU)	0.78 (0.33–1.85)	0.581	0.52 (0.15–1.76)	0.291

The crude odds ratio of the deepest CAL in each subject, P. gingivalis count, and the combination of P. gingivalis counts and P. gingivalis IgG titers were statistically significant. According to the multivariate adjusted odds ratio, only P. gingivalis counts was statistically significant (*p* = 0.034)

*OR* odds ratio, *95% CI* confidence interval with 95% significance level, *PlI* plaque index, *BOP* bleeding on probing, *CAL* clinical attachment level;Counts, each bacteria counts in subgingival plaque, *EU* enzyme-linked immunosorbent assay unit

*p < 0.05

## Discussion

In this study, we evaluated potential markers for periodontitis progression during a 24-month study interval. The results suggested associations between periodontitis progression and bacterial levels in subgingival plaque recovered from the deepest pockets. We previously used PCR to examine the presence of each of six species of periodontopathic bacteria in whole saliva and subgingival plaque [33]. A statistical relationship was found between the presence of *P. gingivalis*, *P. intermedia*, *Prevotella nigrescens*, and *T. denticola* in saliva and in periodontal pocket samples; in the event of disagreement, the organisms were more frequently present in saliva than in periodontal pockets (*p* <0.001). Meanwhile, *A. actinomycetemcomitans* and *T. forsythia* were not reliably detected by sampling of either saliva or periodontal pockets [31].

Our results indicated that The *P. gingivalis* counts of subgingival plaque from the deepest pockets might be used as indicators of the progression of periodontitis. The results of the present study, along with those of our previous study [7], suggest the value of *P. gingivalis* in assessing the progression of CP. Specificity of *P. gingivalis* counts in subgingival plaque from the deepest pockets was higher than that in saliva. The absolute pathogen burden is clearly more informative than merely a dichotomization between carriers and noncarriers [34]. *P. gingivalis*, alone or in combination with *T. forsythia* and *T. denticola*, has been reported to exhibit the deepest mean pocket depths [9]. The thresholds predictive of progressive periodontitis correspond to 0.01% for *A. actinomycetemcomitans*, 0.1% for *P. gingivalis*, and 2.5% for *P. intermedia* [13]. In an 18-month-long study, the presence of *P. gingivalis* and *P. intermedia* in subgingival plaque at proportions exceeding 2% was a significant predictor of periodontitis progression in older adults [35]. These results support the appropriateness using *P. gingivalis* as a marker for the progression of CP, and are consistent with the results obtained in the present study. In the work described here, we evaluated the diagnostic value for periodontal disease of the levels of bacteria in subgingival plaque from the deepest pockets (Table 2), in extension of our previous report [7] showing the diagnostic value for periodontal disease of salivary bacterial levels. Specificity, positive predictive value, likelihood ratio, and AUC of *P. gingivalis* counts for the progression of periodontal disease in subgingival plaque were higher than the respective parameters for bacterial counts in saliva. However, we note that bacterial counts could not be determined in numerous samples of subgingival plaque from the deepest pockets, with detection failing for *P. gingivalis*, *P. intermedia*, and *A. actinomycetemcomitans* in 59.7, 70.2, and 95.7% of samples, respectively.

Subgingival plaque samples from the deepest pockets did not yield detectable levels of periodontal bacteria in several individuals enrolled in this study; however, the salivary samples were able to detect periodontal bacteria in these cases. An inherent limitation of sampling subgingival plaque from the deepest pockets is that these specimens only reflect the condition of sampling site. Notably, however, *P. gingivalis* counts in subgingival plaque from the deepest pockets yielded greater sensitivity and specificity for periodontal progression than did *P. gingivalis* counts in saliva. In this context, it is worth noting that sampling of subgingival plaque from the deepest pockets is more difficult than sampling of saliva. Clinicians using these tests should consider these merits and demerits when considering the use of sampling for periodontal bacteria in subgingival plaque from the deepest pockets. Further investigations will be needed to assess the utility of salivary bacteria levels as markers of periodontal condition.

## Conclusions

The *P. gingivalis* counts of subgingival plaque from the deepest pockets may be associated with the progression of periodontitis.

## Additional file

Additional file 1: List of the ethics committees and approval numbers. (XLSX 12 kb)
